# Optimization and Validation of an In Vitro Standardized Glycogen Phosphorylase Activity Assay

**DOI:** 10.3390/molecules26154635

**Published:** 2021-07-30

**Authors:** Sónia Rocha, Mariana Lucas, Alberto N. Araújo, M. Luísa Corvo, Eduarda Fernandes, Marisa Freitas

**Affiliations:** 1 LAQV-REQUIMTE, Laboratory of Applied Chemistry, Department of Chemical Sciences, Faculty of Pharmacy, University of Porto, 4050-313 Porto, Portugal; up201607090@edu.ff.up.pt (S.R.); mflucas@ff.up.pt (M.L.); anaraujo@ff.up.pt (A.N.A.); 2 Research Institute for Medicines, Faculdade de Farmácia, Universidade de Lisboa, 1649-003 Lisboa, Portugal; lcorvo@ff.ulisboa.pt

**Keywords:** glycogen phosphorylase, optimization, enzyme activity, type 2 diabetes

## Abstract

Glycogen phosphorylase (GP) is a key enzyme in the glycogenolysis pathway and a potential therapeutic target in the management of type 2 diabetes. It catalyzes a reversible reaction: the release of the terminal glucosyl residue from glycogen as glucose 1-phosphate; or the transfer of glucose from glucose 1-phosphate to glycogen. A colorimetric method to follow in vitro the activity of GP with usefulness in structure-activity relationship studies and high-throughput screening capability is herein described. The obtained results allowed the choice of the optimal concentration of enzyme of 0.38 U/mL, 0.25 mM glucose 1-phosphate, 0.25 mg/mL glycogen, and temperature of 37 °C. Three known GP inhibitors, CP-91149, a synthetic inhibitor, caffeine, an alkaloid, and ellagic acid, a polyphenol, were used to validate the method, CP-91149 being the most active inhibitor. The effect of glucose on the IC_50_ value of CP-91149 was also investigated, which decreased when the concentration of glucose increased. The assay parameters for a high-throughput screening method for discovery of new potential GP inhibitors were optimized and standardized, which is desirable for the reproducibility and comparison of results in the literature. The optimized method can be applied to the study of a panel of synthetic and/or natural compounds, such as polyphenols.

## 1. Introduction

**Figure 1:**
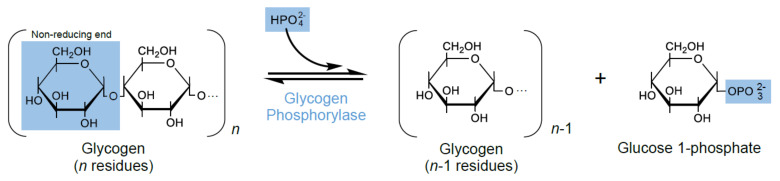
GP reversible reaction, catalyzing the degradation of the non-reducing ends of glycogen into glucose 1-phosphate. HPO_4_^2−^, monohydrogen phosphate anion; OPO_3_^2−^, phosphate group.

Glycogen phosphorylase (GP) (E.C.2.4.1.1) is a key enzyme in the glycogenolysis pathway. Under physiologic conditions and in the presence of inorganic phosphate, GP promotes the catabolic breakdown of glycogen, catalyzing the degradation of α-1,4 glycosidic bonds from the non-reducing end of a glycogen chain, to produce glucose 1-phosphate monomers (Figure 1) [1,2,3]. Despite the fact that GP catalyzes a reversible reaction, the direction of transfer of glucose from glucose 1-phosphate to glycogen is not favorable in physiological conditions due to the relatively high phosphate concentration in the cell [4].

**Figure 2:**
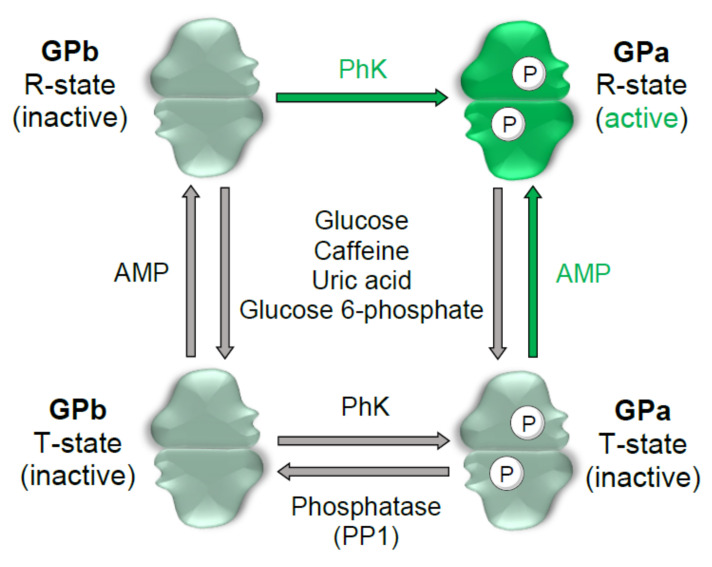
GP activity is modulated by phosphorylation and allosteric interactions. GP occurs as two interconvertible forms, the phosphorylated GPa, and the dephosphorylated GPb. The phosphorylase kinase (PhK) enzyme yields GPa, while dephosphorylation occurs by protein phosphatase 1 (PP1) action. GP also occurs as two conformational states, the inactive T-state (tense state), and the highly active R-state (relaxed state). Through binding at the different GP sites, specific molecule effectors allow transition between the two conformations, either stabilizing the high substrate affinity R-state, or the T-state conformation, with low substrate affinity. Namely, the R-state conformation is stabilized after adenosine monophosphate (AMP) binding to the allosteric site, and glycogen, which binds to an allosteric or to the glycogen storage site. Ligands that stabilize the T-state conformation include glucose, which binds to the catalytic site, caffeine, and uric acid, both with affinity for the inhibitor site, and glucose 6-phosphate that binds to the AMP binding site.

In humans, glucose mobilization benefits from the involvement of three GP isoforms, depending on their localization. In the brain, bGP has a notorious activity alongside energy supply decrements, and in the liver, lGP assures general glucose supply to the further parts of the body from hepatic glycogen stores. In turn, in muscle, mGP activity is pivotal to sustain energy delivery during muscle contraction [5,6]. A high degree of homology (≈80%) in the amino acid sequences was found among the three isoforms [7]. These isoforms are regulated allosterically by diverse molecule effectors and by reversible phosphorylation of serine-14 through the involvement of phosphorylase kinase (PhK), and dephosphorylation by protein phosphatase 1 (PP1). Therefore, it has been described in two interconvertible forms, a phosphorylated form, GPa, and a dephosphorylated form, GPb, but only the phosphorylated form is catalytically active [5,8]. Besides the catalytic center, five other binding sites are prone to external binding, namely the allosteric or adenosine monophosphate (AMP) binding site; new allosteric binding site; inhibitor, purine or caffeine site; glycogen storage site; and the quercetin binding site [2,9]. A schematic illustration of GP regulation is represented in Figure 2.

GP inhibition results in the attenuation of the overproduction of glucose, and for that reason, it is indicated as a potential therapeutic target in the management of type 2 diabetes *mellitus* (DM) [2,6,10]. Type 2 DM is characterized by insulin resistance in skeletal muscle, liver, and adipose tissue, followed by progressive impaired insulin secretion by pancreatic β cells. These abnormities culminate in sustained high levels of circulating glucose, known as hyperglycemia [11,12]. As a complex chronic disorder that requires continuous pharmacological treatments, the control of blood glucose levels remains the most important role for the management of type 2 DM, which is essential to prevent and delay the dire effects of DM [13]. In this respect, the liver has a key role in the regulation of glucose homeostasis, by controlling various pathways in glucose metabolism, namely glycogenesis, glycogenolysis, glycolysis, and gluconeogenesis [3], being gluconeogenesis and glycogenolysis, the main pathways involved in glucose production by the liver [14]. Hence, targeting therapeutics to this organ could foreseeably decrease liver glucose production. However, it has not yet been fully exploited to treat type 2 DM. GP inhibitors, as a novel liver-targeted approach, could be used in combination with other available drug treatments, if additional suppression of circulating glucose is required [15]. A broad number of synthetic and natural origin compounds have been already studied for this purpose [1,2,6,7,8]. However, hitherto, GP inhibitors have not been introduced in the clinic. One important factor is the degree of homology among isoforms. For instance, mGP supports energetic deficits and its inhibition has adverse effects on skeletal muscle. Therefore, finding inhibitors with adequate selectivity to the liver isoform remains a challenge [16].

Caffeine [5,17,18,19] and CP-91149 (5-chloro-*N*-[(1*S*,2*R*)-3-(dimethylamino)-2-hydroxy-3-oxo-1-(phenylmethyl)propyl]-1*H*-indole-2-carboxamide) [16,17] are well-known allosteric inhibitors of GP. Caffeine was the first natural compound with proved binding to the inhibitor site [2]. CP-91149 is known to bind to a novel GP site, different from the caffeine binding site [17]. Some natural polyphenols, such as flavonoids [20,21] and ellagic acid [22], have also been reported as GP inhibitors. Similarly, with caffeine, ellagic acid binds to the inhibitor site of GP [22]. However, it is important to consider that the reported experimental conditions to assess GP activity widely vary between studies, and consequently, make it difficult, or even prevent, the comparison of results among laboratories. For instance, besides the above cited isoforms, other enzyme sources including human, pig, rabbit, and rodent, in both the phosphorylated and dephosphorylated state, thereby generate different results [7]. Since GP catalyzes a reversible reaction (Figure 1) towards glycogen synthesis or breakdown, rather different methods have been additionally described [19]. Moreover, other experimental conditions also differ, including the concentration of enzyme (when GP is used: 1–5 µg/mL) and substrates (when glucose 1-phosphate is used: 0.5–25 mM and glycogen: 1–20 mg/mL), assay temperatures (22–30 °C), incubation times with the compounds under study (no incubation–15 min), buffers (phosphate, imidazole/HCl, HEPES solution, Tris-malate buffers), and pH (6.8 and 7.2) [16,17,18,23,24]. As such, the available data is not always comparable.

**Figure 3:**
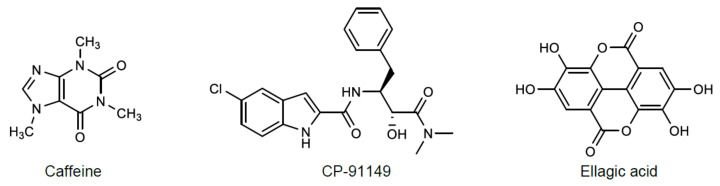
Chemical structures of some known inhibitors of GP activity: caffeine, CP-91149, and ellagic acid.

The present work considers an experimental approach bringing inexpensive and time-saving microanalysis of GP activity. The method can then be broadly implemented for subsequent screening of the inhibitory activity of several compounds, further allowing an accurate and comparable structure-activity relationship study. A method in the direction of glycogen synthesis, which is the most commonly adopted scheme for assays with GP reported in the literature, was selected for optimization. To validate the method, three known GP inhibitors were used, namely a synthetic inhibitor, CP-91149, and two natural compounds, which belong to different families: caffeine, an alkaloid, and ellagic acid, a polyphenol (Figure 3). In order to mimic hyperglycemic conditions, the activity of the most active inhibitor was then evaluated in the absence and presence of high glucose levels. It is crucial to find compounds able to inhibit GP at elevated glucose levels, but with simultaneous lower inhibitory effect when glucose levels are diminished, reducing the risk of hypoglycemia, a common side effect of several current antidiabetic agents [15].

## 2. Materials and Methods

### 2.1. Chemicals

The following reagents were purchased from Sigma-Aldrich, Inc. (St. Louis, MO, USA): dimethylsulfoxide (DMSO), caffeine, CP-91149, ellagic acid, HEPES, MgCl_2_, glucose 1-phosphate, and glycogen. KCl was purchased from José M. Vaz Pereira, S.A. (Sintra, Lisboa). Rabbit muscle GPa was purchased from Creative Enzymes^®^ (Shirley, NY, USA). The reagent for colorimetric phosphate quantitation, BIOMOL^®^ Green, was acquired from Enzo Life Sciences, Inc. (Miraflores, Portugal).

### 2.2. Optimization of the In Vitro Measurement of Glycogen Phosphorylase Activity Assay

**Figure 4:**
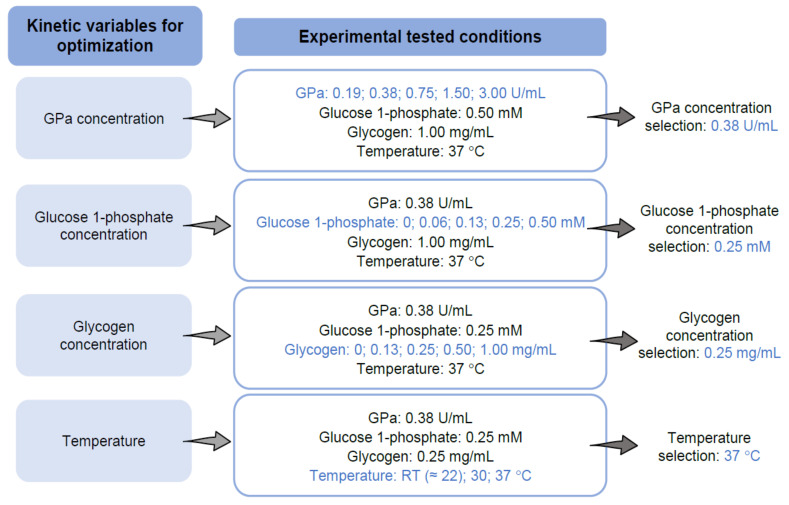
Kinetic variables considered for optimal performance for assessment of the in vitro GPa inhibition assay. RT means room temperature.

The method described by Picot et al. [18], with slight modifications, was followed to settle the most favorable kinetic parameters. To this end, different conditions, as summarized in Figure 4, were screened for the assessment of the in vitro GPa inhibition assay, namely: (1st) GPa concentration, (2nd) glucose 1-phosphate concentration, (3rd) glycogen concentration, and (4th) temperature.

Briefly, and according to Figure 4, the procedure under study was implemented in 96-well plates and started with 50 µL of the rabbit muscle GPa suspension in 50 mM HEPES pH 7.2 solution, at various concentrations (0.19–3.00 U/mL). The enzyme was incubated with 10 µL of DMSO (the solvent of the compounds under study) for 15 min at room temperature (≈22 °C), 30 °C and 37 °C. Following that time period, the catalytic reaction started with the addition of 45 µL of 50 mM HEPES solution, pH 7.2, containing 100 mM KCl, 2.5 mM MgCl_2_, glucose 1-phosphate (0–0.50 mM), and glycogen (0–1.00 mg/mL), and maintained for 30 min at room temperature (≈22 °C), 30 °C and 37 °C. Meanwhile, 130 µL of the reagent for colorimetric phosphate quantitation, BIOMOL^®^ Green, was added to the reaction medium and the absorbance was followed up at the wavelength of 620 nm for 30 min at room temperature (≈22 °C), 30 °C, and 37 °C, using a microplate reader (Synergy HT, BioTek Instruments, Winooski, VT, USA). The blank value (without enzyme) was measured during the assay to identify potential interferences. Each study corresponds to at least four independent assays, results of which are represented graphically as mean ± standard error of the mean (SEM).

### 2.3. Validation of the In Vitro Glycogen Phosphorylase Inhibition Assay

After the optimization of the experimental variables, the inhibitory activity of three different known GP inhibitors was evaluated against GPa activity: caffeine (0–500 µM), CP-91149 (0–3.13 µM), and ellagic acid (0–100 µM). Briefly, 50 µL of the rabbit muscle GPa (0.38 U/mL) was prepared in 50 mM HEPES solution, pH 7.2. The enzyme was incubated with 10 µL of the compounds under study dissolved in DMSO, for 15 min at 37 °C, using 96-well plates. Following that time period, the catalytic reaction started with the addition of 45 µL of 50 mM HEPES solution, pH 7.2, containing 100 mM KCl, 2.5 mM MgCl_2_, 0.25 mM glucose 1-phosphate, and 0.25 mg/mL glycogen, which were incubated for 30 min at 37 °C. Meanwhile, 130 µL of the reagent for colorimetric phosphate quantitation, BIOMOL^®^ Green, was added to the reaction mixture. The obtained results correspond to the absorbance value at 15 min, at the wavelength of 620 nm at 37 °C, using a microplate reader (Synergy HT, BioTek Instruments, Winooski, VT, USA). The results were calculated by subtracting the analytical signal of the blank (without enzyme) to the control value (with enzyme and without the compounds under study) and to the samples’ value. Each study represents at least four independent experiments, results of which are expressed as the mean of the percentage of inhibition of GPa activity ± SEM, or the IC_50_ value ± SEM.

### 2.4. In Vitro Effect of Glucose on Inhibitors’ Potency

Using the optimized experimental settings, the effect of high levels of glucose on the IC_50_ value of the most active GP inhibitor, CP-91149, was investigated. The optimized method was used as described in Section 2.3, using glucose concentrations of 0, 5, and 10 mM added to the buffer that starts the catalytic reaction, 50 mM HEPES solution, at pH 7.2, containing 100 mM KCl, 2.5 mM MgCl_2_, 0.25 mM glucose 1-phosphate, and 0.25 mg/mL glycogen. The results were calculated by subtracting the analytical signal of the blank (without enzyme) to the control value (with enzyme and without the compounds under study) and to the samples’ value. Each study represents at least four independent experiments, results of which are expressed as the IC_50_ value ± SEM.

### 2.5. Statistical Analysis

The results of the in vitro inhibitory activities of the known GP inhibitors, caffeine, CP-91149, and ellagic acid, against rabbit muscle GPa activity, are expressed as the mean of the percentage of inhibition ± SEM or IC_50_ value ± SEM, calculated using GraphPad Prism™ (version 6.0, GraphPad Software, San Diego, CA, USA). The statistical comparisons were performed using one-way analysis of variance (ANOVA) and the differences between groups compared by Tukey’s test, using GraphPad Prism™.

## 3. Results

### 3.1. Optimization of the In Vitro Measurement of Glycogen Phosphorylase Activity Assay

**Figure 5:**
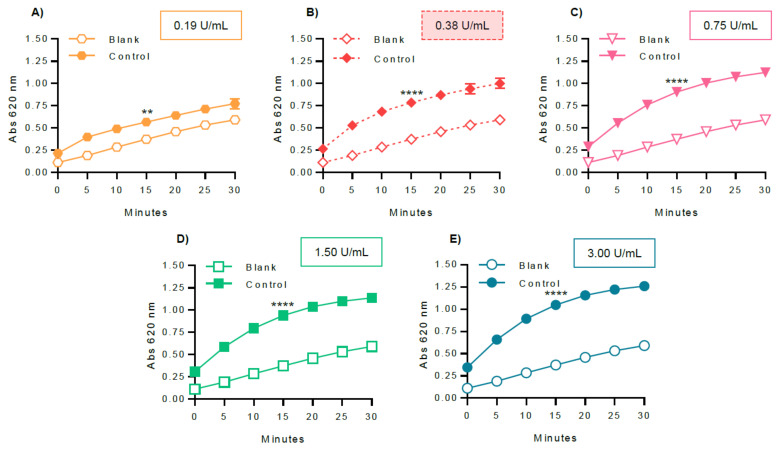
Optimization of the GPa concentration: (**A**) 0.19 U/mL, (**B**) 0.38 U/mL, (**C**) 0.75 U/mL, (**D**) 1.50 U/mL, and (**E**) 3.00 U/mL. Glucose 1-phosphate, glycogen, and temperature were fixed at 0.50 mM, 1.00 mg/mL, and 37 °C, respectively. The blank contained the same constituents as the control, except the enzyme. The obtained results represent at least four independent experiments and were graphically represented as mean ± SEM. ** *p* < 0.01; **** *p* < 0.0001 when compared with the blank (without enzyme) at 15 min of incubation.

**Figure 6:**
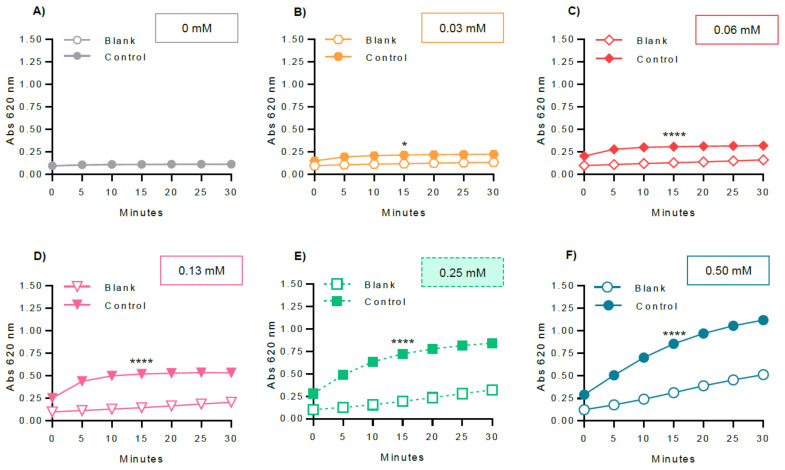
Optimization of the glucose 1-phosphate concentration: (**A**) 0 mM, (**B**) 0.03 mM, (**C**) 0.06 mM, (**D**) 0.13 mM, (**E**) 0.25 mM, and (**F**) 0.50 mM. GPa, glycogen, and temperature were fixed at 0.38 U/mL, 1.00 mg/mL, and 37 °C, respectively. The blank contained the same constituents as the control, except the enzyme. The obtained results represent at least four independent experiments and were graphically represented as mean ± SEM. * *p* < 0.05; **** *p* < 0.0001 when compared with the blank (without enzyme) at 15 min of incubation.

**Figure 7:**
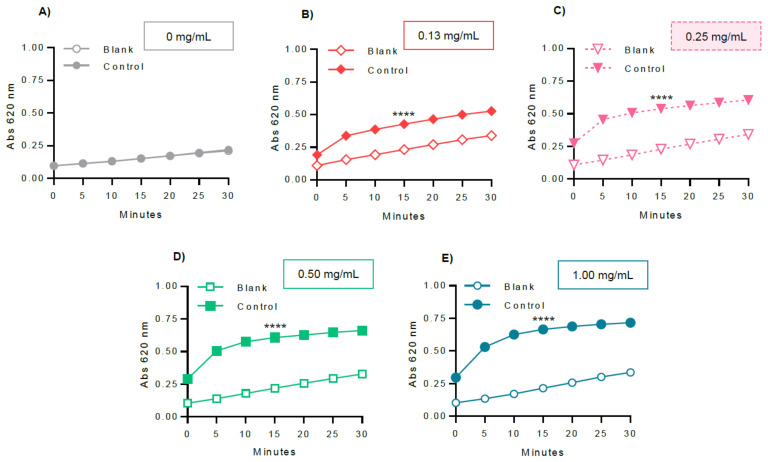
Optimization of the glycogen concentration: (**A**) 0 mg/mL, (**B**) 0.13 mg/mL, (**C**) 0.25 mg/mL, (**D**) 0.50 mg/mL, and (**E**) 1.00 mg/mL. GPa, glucose 1-phosphate, and temperature were fixed at 0.38 U/mL, 0.25 mM, and 37 °C, respectively. The blank contained the same constituents as the control, except the enzyme. The obtained results represent at least four independent experiments and were graphically represented as mean ± SEM. **** *p* < 0.0001 when compared with the blank (without enzyme) at 15 min of incubation.

**Figure 8:**
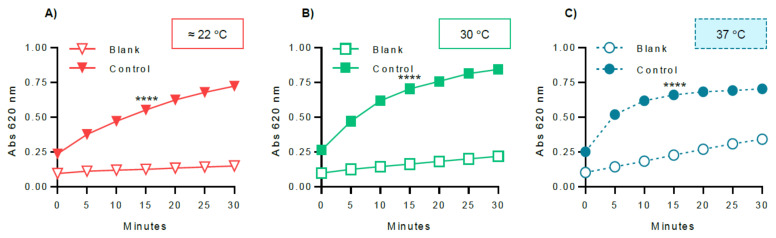
Optimization of the assay temperature: (**A**) ≈22 °C, (**B**) 30 °C, and (**C**) 37 °C. GPa, glucose 1-phosphate, and glycogen were fixed at 0.38 U/mL, 0.25 mM, and 0.25 mg/mL, respectively. The blank contained the same constituents as the control, except the enzyme. The obtained results represent at least four independent experiments and were graphically represented as mean ± SEM. **** *p* < 0.0001 when compared with the blank (without enzyme) at 15 min of incubation.

In order to optimize the experimental variables for GP inhibitory studies, several parameters were evaluated, including GPa amount (Figure 5), glucose 1-phosphate (Figure 6), and glycogen concentrations (Figure 7), and also the dependence of the enzyme activity on temperature (Figure 8). To select the most suitable assay parameters, a statistical comparison between blank (without enzyme) and control values (with enzyme) for each tested parameter was performed using one-way analysis of variance (ANOVA) and the differences between groups compared by Tukey’s test. *K_m_*, the Michaelis constant, is considered as a measure of the enzyme–substrate interaction and reflects the binding affinity between enzyme and substrate [25]. Thus, in order to select the most suitable concentration of glucose 1-phosphate and glycogen, the *K_m_* value, calculated using GraphPad Prism™, based on the Michaelis–Menten model, was also considered. The time of 15 min was selected as the measured point, corresponding to the initial progression of the reaction. Time points extended outside this phase will, in general, correspond to a phase of slowed reaction, due to the depletion of substrates.

The optimization of the in vitro experimental parameters started with the testing of several enzyme concentrations: 0.19, 0.38, 0.75, 1.50, and 3.00 U/mL, by fixing the remaining parameters (0.50 mM glucose 1-phosphate, 1.00 mg/mL glycogen, and temperature at 37 °C), as represented in Figure 5. The ANOVA analysis showed that the concentration of 0.38 U/mL of GPa was the first condition with the highest statistical difference (*p* value < 0.0001) between the blank and control values at 15 min. The analysis also revealed that between the concentrations of 0.38 U/mL and 0.75 U/mL of GPa, no statistical differences were observed. Therefore, the concentration of 0.38 U/mL allowed a suitable enzyme activation and was selected to undertake the following inhibition assays.

Then, the concentration of the enzyme was fixed at 0.38 U/mL, together with 1.00 mg/mL glycogen and temperature at 37 °C, only varying the glucose 1-phosphate concentration between 0, 0.03, 0.06, 0.13, 0.25, and 0.50 mM. The results are represented in Figure 6, and revealed that the concentrations of 0.06, 0.13, 0.25, and 0.50 mM are the ones with the highest significant differences (*p* value < 0.0001) between blank and control values, at the time of 15 min. Additionally, considering that the efficiency of a substrate is represented by its *K_m_* value, this value was further calculated. Accordingly, in this work, the first concentration above the *K_m_* value was selected, which represents a good compromise between substrate concentration and the enzymatic kinetic progress. The observed enzyme kinetic value for *K_m_* was 0.15 ± 0.03 mM. The concentration of 0.25 mM of glucose 1-phosphate was therefore selected for the following assays. Additionally, in order to verify the correct detection time, a non-linear least squares test was applied for the experimental values between 0 and 30 min (see supporting information for more details). The obtained results showed that the parabola vertice corresponds to the least biased results, obtained at the detection time of 15 min.

Once we defined the GPa (0.38 U/mL) and glucose 1-phosphate (0.25 mM) concentrations, the next experimental parameter to be defined was glycogen, by varying the concentration at 0, 0.13, 0.25, 0.50, and 1.00 mg/mL, as represented in Figure 7. Following the same basis, the ANOVA analysis revealed that the concentrations of glycogen at 0.13, 0.25, 0.50, and 1.00 mg/mL showed statistical differences between blank and control values at the time of 15 min (*p* value < 0.0001). Consequently, the *K_m_* value for glycogen was further calculated using GraphPad Prism™. The obtained *K_m_* value for glycogen was 0.21 ± 0.02 mM. Following the same criteria, the first concentration above the *K_m_* value was selected. Therefore, the glycogen concentration of 0.25 mg/mL was chosen to perform the following assays, reaching a significant difference between blank and control values.

The final parameter tested was the variation of the temperature influence on the enzymatic reaction, varying room temperature from (≈22 °C), 30 °C, to 37 °C, fixing GPa at 0.38 U/mL, glucose 1-phosphate at 0.25 mM, and glycogen at 0.25 mg/mL. Room temperature was measured in each experiment to confirm that all experiments were running at 22 °C. The enzyme activities, as a function of the temperature, were measured and the values obtained are represented in Figure 8. The ANOVA analysis showed statistical differences between blank and control values for 22 °C, 30 °C, and 37 °C, at the time of 15 min (*p* value < 0.0001). Additionally, the results at 30 °C and 37 °C demonstrated similar absorbance values at 15 min. It was also possible to observe an increase of the initial enzymatic reaction velocity at the temperature of 37 °C. As 37 °C was the physiological temperature, this temperature was selected to perform the following assays.

### 3.2. Validation of the In Vitro Glycogen Phosphorylase Inhibition Assay

**Figure 9:**
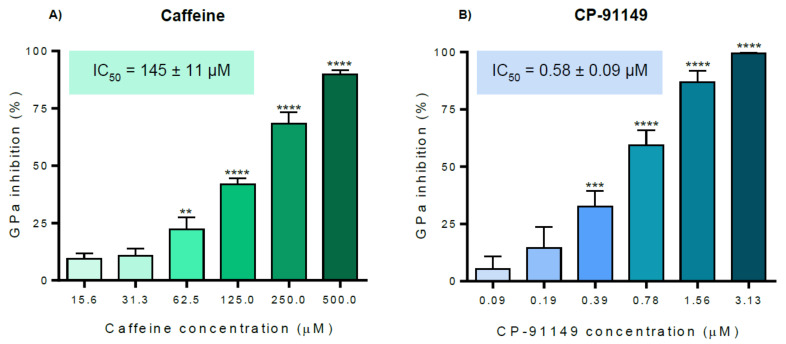
In vitro inhibitory activity of known GP inhibitors, (**A**) caffeine and (**B**) CP-91149, against rabbit muscle GPa. The results are expressed as mean of the percentage of inhibition ± SEM and represent at least four experiments. ** *p* < 0.01; *** *p* < 0.001; **** *p* < 0.0001 when compared with the control (with enzyme and without the compounds under study).

Three known GP inhibitors, namely a synthetic inhibitor, CP-91149 (0–3.13 µM), and two natural compounds, an alkaloid, caffeine (0–500 µM), a polyphenol, ellagic acid (0–100 µM) (Figure 3), were selected for inhibition studies validation using the optimized procedure. The results obtained for caffeine and CP-91149 (Figure 9) enabled us to calculate catalysis inhibition with IC_50_ values of 145 ± 11 µM and 0.58 ± 0.09 μM, respectively. In turn, ellagic acid exhibited an inhibitory activity of 40.6 ± 4.5 % at the highest tested concentration of 100 μM. Comparing the three tested inhibitors, CP-91149 was the most potent inhibitor, being almost 250-fold more effective than caffeine.

### 3.3. In Vitro Effect of Glucose on Inhibitors Potency

To test if the inhibitory activity was regulated by glucose levels, the effect of high levels of glucose on the IC_50_ value of the most active GP inhibitor, CP-91149, was further evaluated, using the optimized ideal experimental parameters. In the absence of glucose, the IC_50_ value of CP-91149 (0.58 ± 0.09 μM) increased when compared with high levels of glucose, 5 mM and 10 mM, which resulted in lower IC_50_ values, 0.39 ± 0.05 μM and 0.22 ± 0.04 μM, respectively.

## 4. Discussion

The implementation of in vitro assays to study enzymatic activities is crucial in drug discovery. Nonetheless, several factors may interfere with a successful assay, ranging from the source of the enzyme and the substrate selection and respective concentrations, adequate assay technology, buffers, reaction conditions, to the equipment handling [26]. The experimental parameters found in the literature to measure GP activity are highly variable, and standardization of these parameters becomes essential to enable a correct reproduction in any laboratory, allowing a comparison of the reported results. Different natural origin and synthetic inhibitors with affinity for different GP sites have been revealed. As an example, most glucose analogs bind to the catalytic center of GP and have demonstrated strong inhibitory activity, with lowering effects on circulating blood sugar in vivo [2]. On the other hand, compounds such as nucleotides, nucleosides, purines, flavonoids, and heterocyclic compounds show a preference for different sites [2]. However, despite the several studies with GP inhibitors, the available data (IC_50_ values or K_i_ values) are not always comparable, due to different experimental conditions. Thus, this work aimed to optimize and validate a screening technique to measure the GP activity to be applied in structure-activity relationship studies and ensure that potential enzyme inhibition is properly estimated.

As GP catalyzes a reversible reaction, different methods for measuring its activity have been employed. Radioactive measurements have been applied, requiring radioactive labeled substrates and special equipment [17,27]. Other authors have implemented methods to measure the glucose 1-phosphate released from glycogen degradation, using electrospray ionization mass spectrometry [28], high performance liquid chromatography (HPLC) [29], or isothermal titration calorimetry (ITC) [19] methods. However, there are two most frequently applied methods in the literature for measuring GP activity. The first method evaluates glycogen breakdown (phosphorolysis). Briefly, in this method, the hydrolysis of glycogen is favored in a coupled enzyme assay, with GP, phosphoglucomutase, and glucose 6-phosphate dehydrogenase. NADPH, generated from glucose 6-phosphate dehydrogenase reaction, is then followed spectrophotometrically at 340 nm. The second method, and most often cited in the literature, evaluates glycogen synthesis, which is based on the colorimetric determination of inorganic phosphate released from glucose 1-phosphate after the glucosyl transfer to glycogen molecule. The first method is not suited for inhibition studies due to potential side interactions between tested compounds and the different enzymes used, phosphoglucomutase and glucose 6-phosphate dehydrogenase [19]. The second method demonstrates relevant advantages, being less expensive due to the use of only one enzyme, and the formed product is easily detected and allows the straightforward investigation of the inhibitory effect of several compounds. Therefore, the second method was selected for this assay optimization. The most frequently used reagent for colorimetric detection of phosphate consists of a mixture of ammonium molybdate and malachite green. Alternatively, a more stable, sensitive, low-cost, and practical method, BIOMOL^®^ Green, was here tested. This reagent provides a time-saving method, since it is a convenient one-step reagent, not demanding mixture with other reagents, and does not require fresh daily preparation, making it ideal for high-throughput applications.

As already mentioned, the amino acid sequence homology among the three GP isoforms (brain, liver, and muscle) is very high (≈80%) [7]. Additionally, the catalytic site is identically conserved in all mammalian GPs, with almost 100% homology for this site, indicating that compounds inhibiting mammalian muscle GP at this site are also able to inhibit human liver GP [10,30]. Thus, in order to use an easily available and low-cost enzyme, avoiding long isolation and purification procedures, the commercial rabbit muscle isoform GP was selected to implement the method. Additionally, among the two interconvertible forms, the phosphorylated form, GPa, and the dephosphorylated form, GPb, the GPa was chosen to perform the present study, since it represents the catalytically active form [5].

After choosing the assay reaction (direction of glycogen synthesis), the reagent for colorimetric detection of phosphate (BIOMOL^®^ Green), and the enzyme (rabbit muscle GPa), several key variables were selected for optimization: enzyme, glucose 1-phosphate, and glycogen concentrations, as well as the assay temperature. In order to select the most adequate parameter, the criteria used in this study were to apply a statistical comparison between each of the tested conditions, also considering the differences between blank and control values, using one-way analysis of variance (ANOVA) and the differences between groups compared by Tukey’s test. As a measure of substrate binding affinity, the *K_m_* value was also considered.

Due to the fact that enzymes are expensive resources, the concentration of the enzyme used in the assay should be as low as possible, but enough to detect the reaction progress, providing an acceptable signal range [25,26]. The literature is highly variable as to the concentration of the enzyme, varying between 1 and 5 µg/mL. In the present experimental study, different concentrations of enzyme were tested: 0.19, 0.38, 0.75, 1.50, and 3.00 U/mL. The concentration of 0.38 U/mL was found adequate to induce a statistically significant difference between blank and control values (*p* value < 0.0001). The statistical analysis also showed that between the concentrations of 0.38 U/mL and 0.75 U/mL of GPa, there were no differences, suggesting that increasing the enzyme concentration to 0.75 U/mL of GPa did not induce a significant effect in the control values.

Besides the enzyme itself, substrates and cofactors and/or coenzymes play a crucial role in enzymatic assays. Different concentrations of glucose 1-phosphate are reported in the literature, varying between 0.5 and 25 mM. In the present study, several glucose 1-phosphate concentrations were tested: 0, 0.03, 0.06, 0.13, 0.25, and 0.50 mM. The statistical analysis showed that the concentrations of 0.06, 0.13, 0.25, and 0.50 mM provided significant differences between blank and control values (*p* value < 0.0001). To select the glucose 1-phosphate concentration, the Michaelis constant *K_m_*, was calculated as a measure for the binding affinity for the substrate, according to Michaelis–Menten equation. The *K_m_* value indicates the concentration of the substrate at half saturation [25]. The *K_m_* value for glucose 1-phosphate was 0.15 ± 0.03 mM. Literature related to the optimal concentration of substrate vs. *K_m_* value to be used in enzymatic assays is not in agreement. Hans Bisswanger [25] advocated the use of concentrations of substrate saturating the catalytic activity of the enzyme, in order to avoid rate limiting conditions. The author reported a 10- to 100-fold concentration of *K_m_*. However, the use of such concentrations of substrate is not always feasible, especially in assays with poorly soluble inhibitors and substrates. Besides, increasing concentrations of substrate may provide an unsuitable absorbance signal [25]. Vicente Sancenon et al. [26] reported substrate concentrations around the *K_m_* as a good compromise for screening or dose-response studies [26]. Angela Proctor et al. [31] reported substrate concentration approximately 3-fold higher than the *K_m_* [31]. Therefore, optimization procedures should be conducted in view of the compromise between substrate concentration and enzymatic kinetic progress to obtain low-cost assays. In this work, 0.25 mM of glucose 1-phosphate, the first concentration above the *K_m_* value, provided a suitable significant absorbance signal and was chosen for the following assays. The increase of the tested glucose 1-phosphate concentrations would result in absorbance values outside the acceptable signal window. The selected value was below the reported concentrations of the literature (0.5–25 mM), allowing reagent-saving. As observed in this optimization step, the blank value was variable among concentrations of glucose 1-phosphate. As such, it was important to follow the enzyme kinetics, in order to understand the role of certain parameters in the assay. In this study, we observed an increase of the blank absorbance signal. This drift could be related to an increase of glucose 1-phosphate concentration, possibly due to the existence of inorganic phosphate as an impurity. This effect can be observed by comparing the blank absorbance values (without enzyme) in the absence of glucose 1-phosphate (0 mM, Figure 6A) with the increasing glucose 1-phosphate concentrations (0.03 mM, 0.06 mM, 0.13 mM, 0.25 mM, and 0.50 mM, Figure 6B–F, respectively). To eliminate this interference, the value obtained for the blank (without enzyme and with glucose 1-phosphate) was subtracted to the value obtained for the control (with enzyme) and samples. Therefore, it is crucial to maintain a significant distance of values between the detected signal of the control and the blank.

Glycogen binds at the glycogen storage site of GP and asserts a regulatory control, as evidenced by the lack of activation of the enzyme in the absence of glycogen (Figure 7A). Compared to other sites of GP, the glycogen storage site has received little attention as a target for inhibitors’ design [2,7,8], possibly because the surface of this region does not suggest potential binding by small molecules exhibiting the desired properties of a suitable drug for oral delivery [7]. The glycogen concentration is also a highly variable parameter reported in the literature, with values varying between 1 and 20 mg/mL. In the present study, several glycogen concentrations were studied: 0, 0.13, 0.25, 0.50, and 1.00 mg/mL. Following the established criteria, the statistical analysis revealed that the concentrations of 0.13, 0.25, 0.50, and 1.00 mg/mL showed significant differences between blank and control values (*p* value < 0.0001). As an essential coenzyme of GPa, the *K_m_* value for glycogen was also obtained: 0.21 ± 0.02 mM. Following the defined criteria, the first concentration above the *K_m_* value was selected. Therefore, the concentration of 0.25 mg/mL of glycogen was chosen to perform the following assays, which enabled a satisfactory absorbance signal. The obtained *K_m_* value was within the range described in the literature, which can vary from less than 0.3 mM to 2 mM, as reviewed by Connett J. and Sahlin K. [32]. Like the glucose 1-phosphate concentration, the selected glycogen concentration was also below the reported concentrations of the literature (1–20 mM), allowing a reagent-saving method.

It is also important to establish a suitable assay temperature to evaluate the enzyme activity. As far as we know, GP assays reported in the literature are performed at room temperature and 30 °C. However, room temperature is not a constant value, and may result in variable results, not only among laboratories, but also depending on several aspects in the same room, including whether doors and windows were open, sunlight radiation was present, or air conditioning used [25]. In this study, three different temperatures were evaluated, the room temperature (measured in every experiment to confirm that all experiments were running at ≈22 °C), an intermediary temperature (30 °C), and the physiologic temperature (37 °C). At room temperature, the activity was evidently lower when compared with that at 30 °C and 37 °C, requiring increasing enzyme concentrations to obtain the same absorbance signal. The results at 30 °C and 37 °C demonstrated similar absorbance values at 15 min. The statistical analysis also showed that between the control values of 30 °C and 37 °C there were no significant differences. However, at 37 °C, it was possible to observe an increase in the enzymatic reaction velocity. In order to mimic the physiological conditions, 37 °C was selected to perform the activation of GPa.

Despite the several studied compounds already reported in the literature, GP inhibitors have not reached the clinic, demonstrating the urgent need to find novel, effective, and safe GP inhibitors. Some reviews cover the patent literature regarding novel structures with inhibitory activity [6,8,10], including CP-91149. In order to validate the implemented procedure, three known inhibitors were tested against GPa, namely a synthetic inhibitor, CP-91149, and two natural compounds, an alkaloid and a polyphenol, caffeine and ellagic acid, respectively. CP-91149 exhibited the highest inhibitory activity. Similar results were obtained by Martin et al. [17], who tested caffeine and CP-91149 against human liver GPa in the glycogen synthesis direction, and obtained an IC_50_ value of 240 and 1 µM, respectively [17]. Freeman et al. [33] tested caffeine against human skeletal muscle GPa in the glycogenolytic direction. Caffeine showed an IC_50_ value of 92 µM [33]. Zhang et al. [16] used CP-91149 as the positive control against rabbit muscle GPa, which exhibited an IC_50_ value of 0.09 ± 0.04 µM, measured in the direction of glycogen synthesis [16]. Kyriakis et al. [22] studied ellagic acid as a rabbit skeletal GP inhibitor in the direction of glycogen synthesis. The authors reported ellagic acid as a potent inhibitor, with *K*_i_s of 7.5 and 13.4 µM for GPa and GPb, respectively [22]. Other authors [20] studied the effect of ellagic acid during glycogen breakdown, which inhibited rabbit muscle GPa and GPb with an IC_50_ value of 3.2 ± 0.5 and 12.1 ± 1.4 µM, respectively. In our experiments, ellagic acid exhibited lower activity at the highest tested concentration of 100 μM.

The differences found in the inhibitory activity of ellagic acid between our work and the results reported in literature could be explained by the different experimental parameters, such as different enzyme (isolated and commercial) and substrate concentrations, and time of interaction between the enzyme and compounds. Therefore, the differences found in the IC_50_ values, reinforce the idea that it is essential to optimize the experimental parameters, in order to allow an accurate reproducibility and comparison of the results among the different studies.

The pharmacological inhibition of liver GP is proposed as a strategy to reduce the overproduction of glucose, limiting the complications of chronic hyperglycemia in type 2 DM [1]. Nonetheless, one of the side effects of the current antidiabetic agents is the risk of hypoglycemia [13]. GP inhibitors may decrease the likelihood of developing hypoglycemia. As previously described [17,23,34], glucose is a physiological regulator of hepatic glycogen metabolism, inhibiting GPa by binding at the active site of the enzyme, stabilizing the T-state conformation. Thus, glucose analogs have been extensively studied as GP inhibitors [2]. However, inhibitors that allosterically bind to the enzyme could be more interesting, because these compounds are reported to be more potent in the presence of high glucose concentrations, acting in a stronger synergistic mode with glucose. Therefore, under high levels of plasma glucose, the characteristic feature of DM patients, these compounds would be more effective, when compared with low or normal levels of glucose. The inhibitory activity could then be regulated by glucose levels, reducing the risk of hypoglycemia, a commonly reported side effect of antidiabetic agents. This characteristic of GP inhibitors provides an attractive clinical profile for drug development [17,23]. CP-91149 was already reported to act synergistically with glucose [17]. To continue the validation of the optimized assay parameters, the effect of high levels of glucose on the IC_50_ value of the most active known inhibitor, CP-91149, was assessed. Our results corroborated the previous results obtained by Martin et al. [17]. The authors reported that at high glucose concentrations, the relative IC_50_ value of CP-91149 decreased by 5- to 10-fold, with an IC_50_ value of 0.13 µM in the presence of 7.5 mM of glucose. In addition, the optimized method was shown to be suitable to study compounds binding at the catalytic site of GP, observed by the synergism with glucose and CP-91149, and the resultant binding of glucose to the active site of the enzyme.

Taking into account that the inhibitory potency of some compounds could be regulated by glucose levels, GP inhibitors are proposed for the management of type 2 DM and related complications, as an antidiabetic target with a low risk of hypoglycemia. Furthermore, they have also been reported as promising for other therapies, possessing cardioprotection (stabilizing cardiac arrhythmias, protection against ischemic injury, survival of transplant hearts) and antitumor properties (preventing tumor growth) [1].

## 5. Conclusions

A high-throughput screening technique to measure GP activity was optimized and validated to be used in the discovery of new inhibitors. The obtained results allowed the choice of the optimal concentration of 0.38 U/mL of GPa, 0.25 mM of glucose 1-phosphate, 0.25 mg/mL of glycogen, and temperature at 37 °C. Three known inhibitors were used to validate the method, CP-91149, as a synthetic inhibitor, and caffeine and ellagic acid, as naturally occurring compounds. The most active inhibitor, CP-91149, was evaluated in the absence and presence of high glucose levels, to mimic hyperglycemic conditions. The potency of CP-91149 increased in the presence of high glucose concentrations, decreasing with lower levels of glucose. Therefore, GP inhibitors are able to act synergistically with glucose, reducing the risk of developing hypoglycemia, a reported side effect of the currently prescribed antidiabetic drugs. GP could be an important target to modulate type 2 DM; as such, this methodology standardization may be fundamental for the reproducibility of the assay among research groups, allowing the discovery of new potential synthetic and natural GP inhibitors.

## Data Availability

The data presented in this study are available on request from the corresponding author.

## Sample Availability

Samples of the compounds are commercially available.
